# Why differentiating between health system support and health system strengthening is needed

**DOI:** 10.1002/hpm.2122

**Published:** 2012-07-09

**Authors:** Grace Chee, Nancy Pielemeier, Ann Lion, Catherine Connor

**Affiliations:** Abt Associates IncBethesda, MS, USA

**Keywords:** health systems strengthening, systems approach, international health, global health initiatives

## Abstract

There is increasing recognition that efforts to improve global health cannot be achieved without stronger health systems. Interpretation of health system strengthening (HSS) has varied widely however, with much of the focus to-date on alleviating input constraints, whereas less attention has been given to other performance drivers. It is important to distinguish activities that *support* the health system, from ones that *strengthen* the health system. Supporting the health system can include any activity that improves services, from distributing mosquito nets to procuring medicines. These activities improve outcomes primarily by increasing inputs. Strengthening the health system is accomplished by more comprehensive changes to performance drivers such as policies and regulations, organizational structures, and relationships across the health system to motivate changes in behavior and/or allow more effective use of resources to improve multiple health services. Even organizations that have made significant investments in health systems have not provided guidance on what HSS entails. While both supporting and strengthening are important and necessary, it is nonetheless important to make a distinction. If activities fail to produce improvements in system performance because they were incorrectly labeled as system *strengthening*, the value of HSS investments could quickly be discredited. Not distinguishing supportive activities from strengthening ones will lead to unmet expectations of stronger health systems, as well as neglect of critical system strengthening activities. Distinguishing between these two types of activities will improve programming impact. Copyright © 2012 John Wiley & Sons, Ltd.

## INTRODUCTION

Recognition that disease-specific interventions implemented in vertical silos are unsustainable has led to growing interest in broader strengthening of health systems. Yet health system strengthening (HSS) has been interpreted in such different ways that there is no common understanding of what HSS really is. When trying to define HSS, many refer to the World Health Organization's (WHO) (2007) framework for describing the functions of a health system. The framework consists of six building blocks: service delivery; health workforce; information; medical products, vaccines, and technologies; financing; and leadership and governance (WHO, 2007). However, these blocks have become synonymous with the health system and with HSS, such that any program making improvements in any block in any fashion can be said to do “health system strengthening.” This ignores that the WHO's (2007) definition of HSS also calls for improving interactions between the building blocks and for sustainable improvements “across health services and health outcomes.”

When launching the Global Health Initiative, US President Obama stated that “We will not be successful in our efforts to end deaths from AIDS, malaria and tuberculosis unless we do more to improve the health systems around the world” (5 May 2009). At this time of heightened interest in, and commitment to, HSS, a clear understanding of HSS is essential.

This paper seeks to clarify the definition of HSS by laying out criteria to distinguish activities that *support* the health system from ones that *strengthen* the health system. S*upporting* the health system can include any activity that improves services, from upgrading facilities and equipment to distributing mosquito nets to promoting healthy behaviors. These activities improve the system's functionality primarily by increasing inputs and can be short term and narrowly focused (for example, distributing free condoms or topping up salaries for target staff for a specified period). In contrast, s*trengthening* the health system is accomplished by more comprehensive changes to policies and regulations, organizational structures, and relationships across the health system building blocks that motivate changes in behavior, and/or allow more effective use of resources to improve multiple health services.

Both supporting and strengthening are important and necessary, and the balance must be driven by the country context. In a fragile, post-disaster, or post-conflict environment, initial efforts should focus on immediate inputs (support) to provide health services, while identifying priority areas for strengthening over the longer term.

The distinction between support and strengthening is important. If activities fail to produce improvements in system performance because they were incorrectly labeled as “health system strengthening,” the value of HSS investments could quickly be discredited. Not distinguishing supportive activities from strengthening ones will lead to unmet expectations of stronger health systems, as well as neglect of critical system strengthening activities. This paper provides criteria to distinguish system *strengthening* activities from system *support* activities, so that the right mix of activities is undertaken to attain both short-term and long-term health goals and ensure that critical HSS needs are not overlooked.

## DIFFERENT APPROACHES TO THE HEALTH SYSTEM

Public health practitioners and development partners approach the health system in many different ways. Some focus on ensuring necessary inputs for the effective delivery of a single or limited set of interventions—vertical programs. This approach has been accused of creating silos and fragmenting the health system. The WHO framework, in contrast, looks across interventions and organizes the health system into its six building blocks or functions.^1^ Although this approach cuts across the vertical disease programs, it too can fragment the health system, albeit by function rather than by disease. The building blocks also further tendencies to segment and limit interventions, under-recognizing the complex and cross-cutting nature of health system constraints. A later WHO publication (de Savigny and Adam, 2009) recognized this constraint, affirming that each of the blocks together do not constitute a functioning health system; instead it is “the multiple relationships and interactions among the blocks—how one affects and influences the others, and is in turn affected by them—that convert these blocks into a system.”

Other approaches to the health system have organized the system by its actors, functions, goals, and strategies (Murray and Frenk, 2000; WHO, 2000; Frenk, 2010; Shakarishvili *et al*., 2010a; van Olmen *et al*., 2010). A comprehensive framework for identifying areas for health system reform was presented by Roberts *et al*. (2004). In their book, the authors describe five “control knobs,” analogous to policy sets, for influencing health sector performance: financing, payment, organization, regulation, and behavior (Roberts *et al*., 2004). This approach focuses on system-wide analysis of policies that affect health system performance, with an emphasis on re-aligning incentives to reward desired behavior.

These various approaches illustrate the wide spectrum of HSS interpretations that currently exists. One reason for the different interpretations is that different actors in the system place priority on different goals—goals that include improved health, responsiveness, social and financial risk protection, and efficiency (WHO, 2007). Another reason is that some disease-specific programs began as an “emergency” response, but the initial emergency approach over time became entrenched. In a true emergency, of course, it is entirely appropriate to use helicopters to fly in medical supplies; over time, however, it is more effective to repair the road and improve the distribution system. There is increasing recognition of the tradeoff between applying immediate solutions that do not strengthen the existing infrastructure/system and making investments that may take longer to reap health benefits, but are more likely to have a lasting impact. Both approaches are important, and must be balanced appropriately, to achieve global health and development goals.

## WHAT IS HEALTH SYSTEM STRENGTHENING?

Quite simply, health system *strengthening* is about permanently making the system *function* better, not just filling gaps or *supporting* the system to produce better short-term outcomes. The WHO definition of HSS (WHO, 2007) fully supports this concept:

‘improving [the] six health system building blocks and managing their interactions in ways that achieve more equitable and sustained improvements across health services and health outcomes’

The definition has been interpreted much more narrowly, however, stopping at “improving the six health system building blocks” to characterize vertical programs as HSS. This overlooks managing the *interactions* between and among the building blocks, and the call for *equitable and sustained* improvements *across* health services.

In its entirety, the WHO definition encompasses true HSS. What is needed is not an alternative definition but a way to depict more limited health system *support* activities from more holistic health system *strengthening* activities.

Human health provides a useful analogy to illustrate the difference between supporting and strengthening the system. Consider a person who upon a physical exam is found to be overweight and have high blood pressure. A simple response prescribes blood pressure medication to reduce blood pressure and its associated risks relatively quickly. This response is analogous to a system *support* intervention. While it addresses the direct problem, it does not actually improve the individual's health, and his high blood pressure will return once medication is stopped. In contrast, system strengthening responses would entail weight loss, improved diet, and exercise—all of which fundamentally improve health. These activities require a longer-term investment, and more active commitment from the individual, but ultimately produce results that may lead to the end of support (blood pressure medication) and make the system stronger in other ways—improving respiratory function, immune response, overall energy levels, and so on. This analogy also illustrates the importance of balancing both supportive and strengthening activities simultaneously.

## THE HEALTH SYSTEM VIEWED AS A CUBE

**Figure 1:**
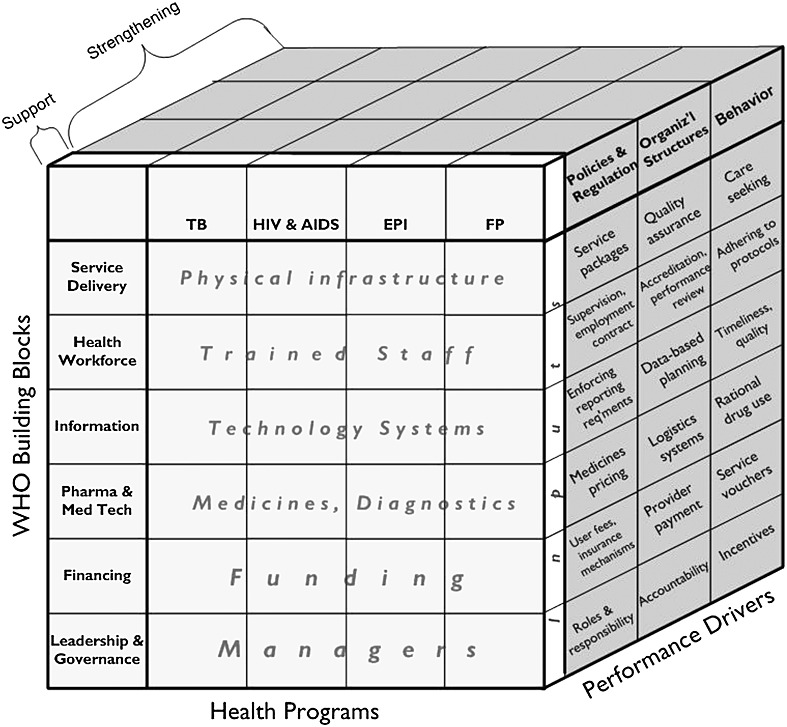
The health system cube

We propose to depict the distinction between system *support* from system *strengthening* with a three-dimensional cube (Figure 1). The cube shows the *WHO building blocks* that define the core functions of the health system, illustrative *disease-specific programs* that deliver critical services, and sets of *performance drivers*.^2^ The performance drivers include inputs, as well as policies and regulations, organizational structures, and behaviors that affect how well those inputs are used to produce outcomes (Roberts *et al*., 2004). These are shown in the third dimension, with illustrative examples.

Adding this third dimension encourages more in-depth analysis of the multiple facets of the building blocks that affect performance. Improved performance can be driven by increasing inputs or by improving the use of those inputs. As has been noted, the focus to date has been on the front slice of the cube—the inputs. Although those inputs are necessary, they alone do not produce lasting change. Permanent performance improvement requires delving deeper into the system to change policies, structures, and behaviors that can improve the way inputs are used—to improve equity, access, quality, and efficiency.

The health system cube helps to distinguish health system *support* activities from system *strengthening* activities. An intervention to *strengthen* the system goes beyond providing inputs (it must have depth) and applies to more than one health program (it must have breadth). Although the cube cannot illustrate all the interactions between the building blocks, nor the factors beyond healthcare provision that also affect health outcomes, it captures the distinction between health system *support* and health system *strengthening*.

This cube allows us to appreciate the depth of each building block. Take, for example, the health workforce: although production of trained workers (a workforce input) is an obvious driver of workforce performance, it is not the only driver. Workforce distribution, compensation, performance monitoring, worker behavior, and other factors also contribute to a well-performing health workforce. In practice, however, training is not supported with policies and practices to ensure application of new skills, retain skilled workers, or motivate high performance. The reauthorization of PEPFAR 2 is an example of the focus on inputs; its sole “health system strengthening” indicator is the training of 140 000 healthcare workers.

Admittedly, it is easier to identify gaps in inputs and address them in isolation than to analyze the more complex interrelationships underlying the health system. Using the example of the workforce, it is relatively easy to train more workers, but more challenging to design and implement policies and activities related to employment conditions and contracts, staff placement, or performance incentives that address bottlenecks such as inadequate staffing or low motivation. These tasks require support from other ministries or collaboration with sub-national government authorities. These issues are also intricately related to other system building blocks: the quality of service facilities and availability of supplies and equipment affect staff morale; clear roles and responsibilities, and effective accountability mechanisms affect staff performance; and salary policies affect staff retention. Despite their complexity, these issues must be addressed if the goal is a stronger health system.

Building upon the concepts illustrated by the cube, we developed a set of four criteria, shown in Box 1, for assessing what is and is not HSS.

1. The interventions have cross-cutting benefits beyond a single disease. Regardless of the ultimate goals, an intervention cannot be considered HSS if it does not strategically provide benefits beyond a single disease or intervention. For example, whereas functioning cold chain equipment is an absolute requirement for the EPI program, investments in cold chain support the EPI program, not the health system. Creating a more cohesive and integrated health system (in financing, service delivery, and so on) and reaching across HIV/AIDS, MCH, and other services is one of the core strategies for improving health systems.
2. The interventions address identified policy and organizational constraints, or strengthen relationships between the building blocks. Although more resources are helpful in supporting weak health systems, health systems do not necessarily function better with the infusion of more inputs. Strengthening the system requires revising policies and improving relationships to overcome constraints and bottlenecks. Buying generators for health facilities *supports* the health system. *Strengthening* the system might include creating a mechanism to conduct regular surveys of facility equipment (possibly identifying generators as requiring replacement), complemented with revised budgeting procedures that include funding for equipment maintenance and replacement. This latter intervention does not just provide an input but also changes budgeting procedures and strengthens relationships between the system's finance, supplies and equipment, and service delivery functions.
3. The intervention will produce long-term systemic impact beyond the life of the activity. This criterion is best explained with an example regarding staff training. Health worker training is often proposed as an HSS activity with lasting impact. Although trained health workers do apply their skills after the training period, providing training to address the current skills gap is a short-term solution. The same health workers will require retraining, whether in 1 or 8 years, whether in the same skills or others, and there will be no mechanism for providing that training. Strengthening the health system so that health workers can be well trained involves allocating a training budget, creating a periodic procedure for conducting skills assessments, establishing mechanisms to regularly review training curricula, and creating institutional capacity to conduct training on an ongoing basis. Although resources might not be sufficient to pursue all these activities, at least some of these measures must be pursued to distinguish a system-strengthening activity from a system–support activity.
4. The interventions are tailored to country-specific constraints and opportunities, with clearly defined roles for country institutions. There is no perfect model for health system strengthening because it must respond to the country context (e.g., introducing health insurance may or may not be the highest priority for improving financing in all countries). Effective HSS interventions should target constraints that can have maximum benefit across various health programs. The second component of the criterion above addresses a common constraint across all weak health systems: weak leadership and vision at multiple levels of the system. One could argue that stronger leadership is the answer to all the system weaknesses, but creating a health system with leaders does not come overnight or from a training program. It requires roles for government institutions that make full use of, and build upon, existing leadership capacity regardless of the country context or the HSS interventions (whether strengthening NGOs, or improving HIS, for example). HSS should always reinforce and strengthen the leadership role of government health authorities.

**Box 1: Is it Health System Strengthening?**

- Do the interventions have cross-cutting benefits beyond a single disease?
- Do the interventions address policy and organizational constraints or strengthen relationships between the building blocks?
- Will the interventions produce permanent systemic impact beyond the term of the project?
- Are the interventions tailored to country-specific constraints and opportunities, with clearly defined roles for country institutions?

Interventions must meet all these criteria in order to make long-term improvements to health systems. A basic outcome difference between health system *strengthening* and *support* is that whereas providing support addresses the constraints currently found, strengthening the system actually changes the system so that it can address these constraints in the future. A stronger health system is more able to adapt and respond to external changes, whether emerging diseases, financial crises, or population migrations. A weak health system is often static—it is unable to recalibrate drug procurement and distribution even though some drugs are always out of stock whereas others are overstocked or to reassign staff even though some facilities are understaffed whereas others are overstaffed. In contrast, a strong health system is dynamic and able to respond to changes independently. Although *supporting* the system alone can improve performance in the short term, only activities that go beyond to *strengthening* the system can improve system's ability to respond to future challenges.

## ARE WE SUPPORTING OR STRENGTHENING?

Applying the distinction between supporting and strengthening developed in this paper, we find that many activities currently undertaken and labeled as HSS support the system, but do little to strengthen it. Other publications also question whether HSS activities *strengthen* systems, as well as note the general lack of operational understanding of HSS.

A GAVI Alliance-commissioned evaluation of its HSS funding found that countries used funding for interventions focused on “immediate support to deliver” immunization and maternal and child health services (HLSP, 2009). Generally, the activities GAVI funded fell within the front slice of inputs required to *support* service delivery. Another analysis of GAVI HSS funding (Goeman *et al*., 2010) found that countries primarily proposed “short-term operational responses, rather than more complex, longer-term approaches to health system strengthening.” On the basis of the study's classification of activities as “operational” or “systemic,” with operational activities defined as those that do not involve comprehensive change at a higher, systemic level, the authors found that 17% of the budget was allocated toward “systemic” activities (Goeman *et al*., 2010).

The Global Fund to Fight AIDS, Tuberculosis and Malaria and its recipients have also struggled to define HSS and address health system weaknesses (Sherry *et al*., 2009). In the first round of funding for HSS, only three out of 30 countries were approved, with the Global Fund Technical Review Panel (GFTRP) (2005) reporting that “the definition of HSS…was too vague and too broad.” In more recent reports, the GFTRP (2006) has also highlighted potential negative impacts on other parts of the health system of HSS proposals seeking to address human resource issues by recruiting new staff and paying salary premiums, as well as concerns (GFTRP 2008) that the WHO building blocks approach may “dissuade rather than incentivize stronger responses to what are undoubtedly complex, integrated bottlenecks to the delivery…of improved equity, efficiency, and quality in their health systems.” More guidance is now provided to define HSS activities, but the GFTRP (2009) noted in Round 9 that “many applicants are often requesting a ‘shopping’ list of all theoretical HSS needs.”

Lack of a common understanding of HSS causes frustration for countries, funding organizations, and advocates of holistic health system approaches. The GAVI Alliance, Global Fund, World Bank, and WHO are working together within a joint HSS platform; however, there has been more focus on processes for harmonization and alignment than on common understanding of HSS. A recently published paper (Shakarishvili *et al*., 2010b) proposing a framework for HSS resource tracking suggests prerequisites for a common framework, including “harmonization of conceptual and operational understanding of HSS” and “agreement on inclusion/exclusion criteria for HSS expenditures.” There is clear recognition of differences in understanding, and need for coherence. In the current funding-constrained environment, better understanding is needed to ensure continued support of HSS.

## CONCLUSIONS

The need to distinguish between health system *strengthening* and *support* activities is not an academic one. It is important to increase understanding of the scope of HSS, so that countries, donors, and implementers can look beyond identifying the input gaps to identifying the policy and structural constraints that impede better performance. Activities currently undertaken as HSS address many important gaps, but overlook other systemic problems that need to be addressed to improve global health. The risk of the current confusion that undertakes system *support* activities in the name of system *strengthening* is that ultimately these activities will not produce long-term improvements in the health system, and system strengthening may be discarded as ineffective. Both *support* and *strengthening* are needed, but clarity about the differences will improve programming impact.

We hope that the criteria proposed here can be the basis for discussion in debates of how to design more effective system *strengthening* interventions that have long-term impact on health services. Within donor and other funding organizations, we hope that these criteria can spark discussions regarding how best to allocate resources between *support* and *strengthening* activities, in line with development goals. Creating a distinction between these two types of activities that is understood by all stakeholders will improve design and evaluation of health system *strengthening* activities, resulting in better-performing health systems and ultimately permanent improvements across all health services and health outcomes.
